# Evaluation of dsRNA produced in *E. coli* for controlling Japanese beetle

**DOI:** 10.3389/finsc.2026.1811042

**Published:** 2026-04-29

**Authors:** Jeffrey L. Howell, Ramesh Kumar Dhandapani, Sundararajan Balasubramani, Jun Seok Ryoo, Subba Reddy Palli

**Affiliations:** Department of Entomology, College of Agriculture, Food and Environment, University of Kentucky, Lexington, KY, United States

**Keywords:** double-stranded RNA, gene silencing, Japanese beetle, pest management, RNAi

## Abstract

The Japanese beetle (*Popillia japonica*) is a highly polyphagous invasive pest that causes significant economic damage to field crops, ornamentals, and turf. Traditionally, large amounts of broad-spectrum chemical insecticides have been used to target both adults and larvae, but with limited success mainly due to resistance. RNA interference (RNAi) might provide an alternative method for species-specific control by silencing essential survival genes with double-stranded RNAs (dsRNAs). Injecting dsRNA targeting the actin and ATPCL genes reduced their expression and caused death in Japanese beetle adults. However, orally delivered dsActin did not induce knockdown of the target gene or mortality. Exposure of dsRNA to lumen contents, followed by electrophoresis, showed that dsRNases present in the lumen digest dsRNA, suggesting that degradation of orally delivered dsRNA may contribute to its ineffectiveness. In contrast, dsActin produced in *Escherichia coli* and fed to adult beetles caused target gene knockdown, reduced feeding on rose leaves, and increased mortality. When heat-killed bacteria were sprayed onto okra plants in covered field plots, a similar effect was observed; the plants were better protected by dsActin than by control dsGFP. These results from laboratory assays and preliminary field trials suggest that using heat-killed bacteria that produce dsRNA targeting essential genes in Japanese beetles could offer a cost-effective strategy for its control.

## Introduction

The Japanese beetle [*Popillia japonica* (JB)] is native to Japan, but since its introduction to the United States in 1916, it has become a rampantly invasive and damaging pest, particularly in the eastern states (1). The JB is responsible for major economic losses exceeding $450 million annually, resulting from targeted management and damage mitigation. This highly polyphagous insect feeds as an adult on the foliage, flowers, and fruits of over 300 plant species, while subterranean larvae feed on grass roots, severely damaging lawns and fields in infested areas (2). The JB is a serious concern and is the target of interstate and international quarantine efforts on nursery plants and at airports in some states where it is established. In the early 1970s, the JB was accidentally introduced to the Azores (Portugal) from a US air base and has recently been detected in northern Italy, where it is actively spreading. Indeed, there is concern that the JB will become a prominent invasive pest throughout much of temperate Europe due to favorable climatic conditions and its capacity to feed on such a broad range of host plants (2–4). Early detection, quarantine, and eradication are the primary strategies for combating JB. Some traditional residual insecticides (e.g., carbaryl) can be effective in controlling adults when applied to leaves and flowers, but their nonspecific nature can harm beneficial species and the environment. Because of the costs, challenges, and environmental concerns associated with chemical treatment of large numbers of host plants and infested areas, there is an immediate need to develop novel control methods for this invasive pest (5).

RNA interference (RNAi) is being developed as a highly targeted alternative to chemical insecticides for insect pest management (6, 7). RNAi is a post-transcriptional gene-silencing mechanism initiated by the introduction of double-stranded RNA (dsRNA). RNAi was first discovered in the nematode *Caenorhabditis elegans* and has since been observed in humans, plants, and insects (7). RNAi technology has emerged not only as an extremely powerful tool for functional genomics studies but also as a potentially useful method for pest control. The first commercial RNAi-based pest-control method uses transgenic corn plants to deliver dsRNA targeting an essential gene encoding the vacuolar sorting protein SNF7 to the Western Corn Rootworm (8). Two additional products to control Colorado potato beetles (9) and varroa mites (https://downloads.regulations.gov/EPA-HQ-OPP-2023-0558-0086/content.pdf) in honey bee hives have been registered.

dsRNA targeting essential genes in *Caenorhabditis elegans*, produced in *Escherichia coli, was shown to induce RNAi* (10). In insects, the same strategy was applied in *Helicoverpa armigera* (11), *Leptinotarsa decemlineata* (12), *Spodoptera exigua* (13), *Chilo suppressalis* (14) and *Epiphyas postwittana* (15). Recently, Carroll et al. (16) showed that dsRNA targeting peritrophin in JB complexed with branched amphiphilic peptide capsules induced knockdown of the target gene and caused 40-50% mortality of adults. We aimed to identify potential target genes for RNAi-mediated pest control of JB and to test their efficacy in inducing mortality in both laboratory and field settings. Six target genes were screened in dsRNA injection assays, and the actin gene was identified as the most promising candidate for RNA-mediated control of this pest. We then expressed dsRNA targeting the actin gene in *E. coli* and showed that feeding JB adults with heat-killed bacteria containing dsActin induced target gene knockdown and mortality in laboratory bioassays. In preliminary field trials, dsActin-expressing heat-killed bacteria protect okra plants from JB adult damage in covered plots.

## Materials and methods

### Insect collection and rearing

The adult JB used in this study were field collected by hand from the University of Kentucky’s organic south farm in Lexington, Kentucky. The beetles were maintained in BugDorm-1 screen cages inside an incubator chamber at 23 ± 2 °C, 80-90% relative humidity, and a photoperiod of 14 hr light/10 hr dark. The beetles were provided with fresh linden leaves (*Tilia cordata* Mill.) for 24 hr after collection, then starved overnight before conducting assays the following day.

### *In vitro* dsRNA synthesis

Fragments of target genes were amplified using gene-specific primers with T7 polymerase promoter sequence (TTAATA CGACTCACTATAGGG) at their 5′ ends (**Supplementary Table 1S**). Six target genes were selected for screening: Actin, ATPCL, SSK, SNF7, Sec23, and IAP. Candidate genes for testing as RNAi targets were selected based on those reported to be effective in published studies (16–19). Total RNA was extracted from Japanese beetles using Trizol reagent. Two μg of RNA was used to synthesize cDNA. The resulting cDNA was combined with a 2x PCR master mix and amplified under the following conditions: 94 °C for 4 minutes, then 35 cycles of 94 °C for 30 seconds, 58 °C for 30 seconds, and 72 °C for 45 seconds, with a final extension at 72 °C for 10 minutes. The PCR product was purified using the Qiagen PCR purification kit (Qiagen Inc., Valencia, CA, USA). As a control, a fragment of the gene encoding green fluorescent protein (GFP) was used. The PCR products served as templates for dsRNA synthesis using the MEGAscript RNAi Kit (Ambion Inc., Foster City, CA, USA). Twenty nanograms of purified DNA were used in a 20-μl reaction. The reaction was incubated for 16 hours at 37 °C, then treated with DNase I for 30 minutes. The dsRNA was precipitated by adding 0.1× volume of 3 M sodium acetate (pH 5.2) and 2.5× volume of 100% ethanol, followed by incubation at −20 °C for 2 hours and centrifugation at 4 °C for 15 minutes. The dsRNA pellet was rinsed with 750 μl of 75% ethanol and centrifuged again at 4 °C for 5 minutes. After removing the ethanol, the pellet was dried and dissolved in ultrapure distilled water. The quality of the dsRNA was verified by 1% agarose gel electrophoresis and quantified using a spectrophotometer (NanoDrop Technologies, Wilmington, DE, USA) as previously described (20).

### dsRNA injection bioassays

To identify efficient target genes for RNAi-mediated control of JB, 10 adults were injected with 10 µg of dsRNA per target gene. After injection, adult beetles were maintained individually on freshly cut linden leaves inside 16-oz plastic deli containers with screen-vented lids. All treated insects in their rearing containers were maintained in an incubator under standard rearing conditions, and mortality was recorded on day 10 after injection.

### Expression of dsActin in *E. coli*

A recombinant plasmid vector for dsRNA expression corresponding to an Actin and GFP gene fragment was constructed. The purified PCR product was digested with a restriction enzyme and inserted into the L4440 vector. The L4440 vector contains two T7 RNA polymerase promoters flanking multiple cloning sites. The recombinant plasmid L4440-Actin was transformed into competent *E. coli DH10B* cells. The positive clone was confirmed by colony PCR, and plasmid DNA was isolated using a plasmid mini preparation kit (Qiagen) according to the manufacturer’s instructions.

### Preparation of dsRNA in bacteria and feeding bioassays

*In vitro* transcription is expensive; therefore, synthesizing large quantities of dsRNA required for experiments or field applications involving insect bioassays is not cost-effective. Hence, dsRNA synthesis in RNase III-deficient bacterial cells was attempted. HT115 (DE3) is a strain of *E. coil* that has been genetically engineered to enable dsRNA synthesis. HT115 (DE3) is a genetically engineered strain of bacteria that is deficient in RNase III. Competent HT115 (DE3) cells were prepared, and the plasmids L4440-Actin and L4440-GFP were transformed into competent cells. dsRNA synthesis and isolation were performed as previously described (10). Transformed HT115 (DE3) cells were inoculated into Luria-Bertani (LB) medium and cultured overnight at 37 °C. The culture was diluted 1:100 and grown to an OD600 of 0.4–0.6. Isopropyl β-D-1-thiogalactopyranoside (IPTG) was added to a final concentration of 0.4 mM, and the culture was incubated with shaking at 37 °C for 4 hr After 4 hr, the cells were harvested by centrifugation, and total nucleic acids were extracted. The expressed dsRNA was confirmed by 1% agarose gel electrophoresis. After dsRNA-induced overexpression, the culture broth was centrifuged at 2500 rpm for 10 min, and the supernatant was removed. The pellet was washed with 1×PBS by centrifugation. The dry pellet was then dissolved in 2 mL of sterile water. The bacteria were heat-killed at 80 °C for 30 min. Prior to feeding, bacterial viability was assessed by plating 100 µl of the heat-killed bacterial sample onto LB agar plates supplemented with ampicillin and tetracycline.

### Naked dsRNA feeding bioassays

To test the insecticidal efficacy of naked dsRNA against the JB, linden (*Tilia cordata Mill.*) leaves were coated with naked dsRNA (300 µg) targeting dsActin or dsGFP (Control) and then fed to adult beetles. After the leaves dried, 10 adult Japanese beetles were released and allowed to feed freely on the treated plant material. The leaf material was refreshed, and mortality rates were observed daily for 15 days. For the knockdown assay, adult beetles were collected after five days of feeding for qPCR analysis.

### Stability of dsRNA in the midgut lumen

To study the degradation of dsRNA by the nuclease present in midgut lumen contents, a previously reported protocol (17) was used. JB adult lumen contents were collected in 200 µl of 1X phosphate-buffered saline (PBS) and centrifuged for 10 min at 13000 rpm at 4 °C. The supernatant was collected and centrifuged again at the same speed. One microgram of dsRNA was incubated with one microgram of midgut lumen contents at room temperature. Samples were collected at 1, 2, 3, 6, 12, and 24 hr and stored at -20 °C. The samples were run on a 1% agarose gel, stained with GelRed, and photographed using an iBright™ CL1500 Imaging System (ThermoFisher Scientific, USA).

### Adult feeding bioassays

To test the insecticidal efficacy of bacterially expressed dsRNA against the Japanese beetle, rose plant (*Rosa* sp.) leaves were sprayed with inactivated bacteria expressing dsRNA targeting dsActin or dsGFP (control), and the leaves were then fed to adult beetles. Single stems of either linden or rose, approximately the same size, were placed in a small flask containing 50 mL of water and sealed with Parafilm around the stem. A total of 2 mL of inactivated bacteria was evenly coated on each leaf of the stem (9 leaves per linden stem, 20 leaves per rose stem). After the leaves dried, 10 adult Japanese beetles were released and allowed to feed freely on the treated plant material. The insect-feeding bioassay was performed under controlled conditions at 23 ± 2 °C, 80-90% relative humidity, and a photoperiod of 14 h light/10 h dark. The leaf material was refreshed, and mortality rates were observed daily for 7 days. For the knockdown assay, adult beetles were collected after three days of feeding for qPCR analysis.

### Quantitative real-time PCR

Total RNA was extracted using TRIzol reagent. Two micrograms of total RNA were used for first-strand cDNA synthesis with M-MLV reverse transcriptase (Invitrogen, USA). This cDNA was used as a template for RT-qPCR. Each RT-qPCR reaction contained 5 µl of iTaqTM universal SYBR Green Master Mix, 2 µl of 1:3-diluted cDNA, and 0.5 µl each of 10 µM forward and reverse gene-specific primers (**Supplementary Table 1S**). Amplification conditions were as follows: 95 °C for 20 s, followed by 40 cycles of 95 °C for 5 s and 60 °C for 20 s. At the end of each RT-qPCR reaction, a melting curve was generated to confirm a single peak and rule out primer-dimer and non-specific product formation. RPL32 was used as a reference gene, and the 2^−ΔΔCt^ method was used to calculate the target gene’s relative expression in the samples relative to controls.

### Statistical analysis

Statistical significance of expression levels was determined by one-way ANOVA with Turkey *post hoc* analysis. A p-value < 0.05 was considered significant for differences between the control and treatment groups.

### Field studies

Okra (Jambalaya) was grown in greenhouse conditions before being transplanted to a designated plot at the University of Kentucky’s South Farm. The okra was planted with 2-foot spacing in four rows, each containing 80 plants. Prior to spraying and beginning the experiment, all okra fruits were removed from every plant in the plot. All weeds were cleared from each plot before the experiment commenced and prior to any follow-up application.

Two plots, each containing 10 plants, were enclosed in the field using 8-foot rebar stakes and 14-foot-wide fine-mesh horticultural netting (ProtekNet) to prevent insects from entering or escaping. These plots were treated every week with either heat-killed bacteria expressing dsGFP or dsActin. The solutions also contained a 1:100 volume ratio of two adjuvants: NuFilm P (spreader-sticker) and Monterrey Horticultural Oil (penetrator). Treatments were applied with a gas-powered sprayer at approximately 50 mL per plant. 24 hr after the first application, 50 female JBs were released into each enclosed plot on July 24, 2023. An additional 50 female beetles were released into each plot on August 7, 2023.

Five additional plots of 10 plants each were randomly selected for the other treatments, including Carbaryl (positive control), heat-killed bacteria expressing dsGFP, heat-killed bacteria expressing dsActin, heat-killed bacteria expressing dsActin with adjuvants, and an untreated control plot. Starting on August 18^th^, Carbaryl was replaced with Talstar P (Bifenthrin) as a positive control because Carbaryl-treated plots showed limited protection. Foliage damage and beetle densities were estimated weekly by two observers on each plot.

Leaves were collected 24, 48, 72, 96 hr, and 1 week after spraying for dsRNA stability analysis. These samples were stored in Trizol in a -80 °C freezer. When JB populations were no longer detectable in the field, the five top leaves from each plant were collected and analyzed to quantify damage. Okra from each plot was harvested, and the weight was recorded.

## Results and discussion

### Screening target genes by microinjection assay in JB adults

**Figure 1:**
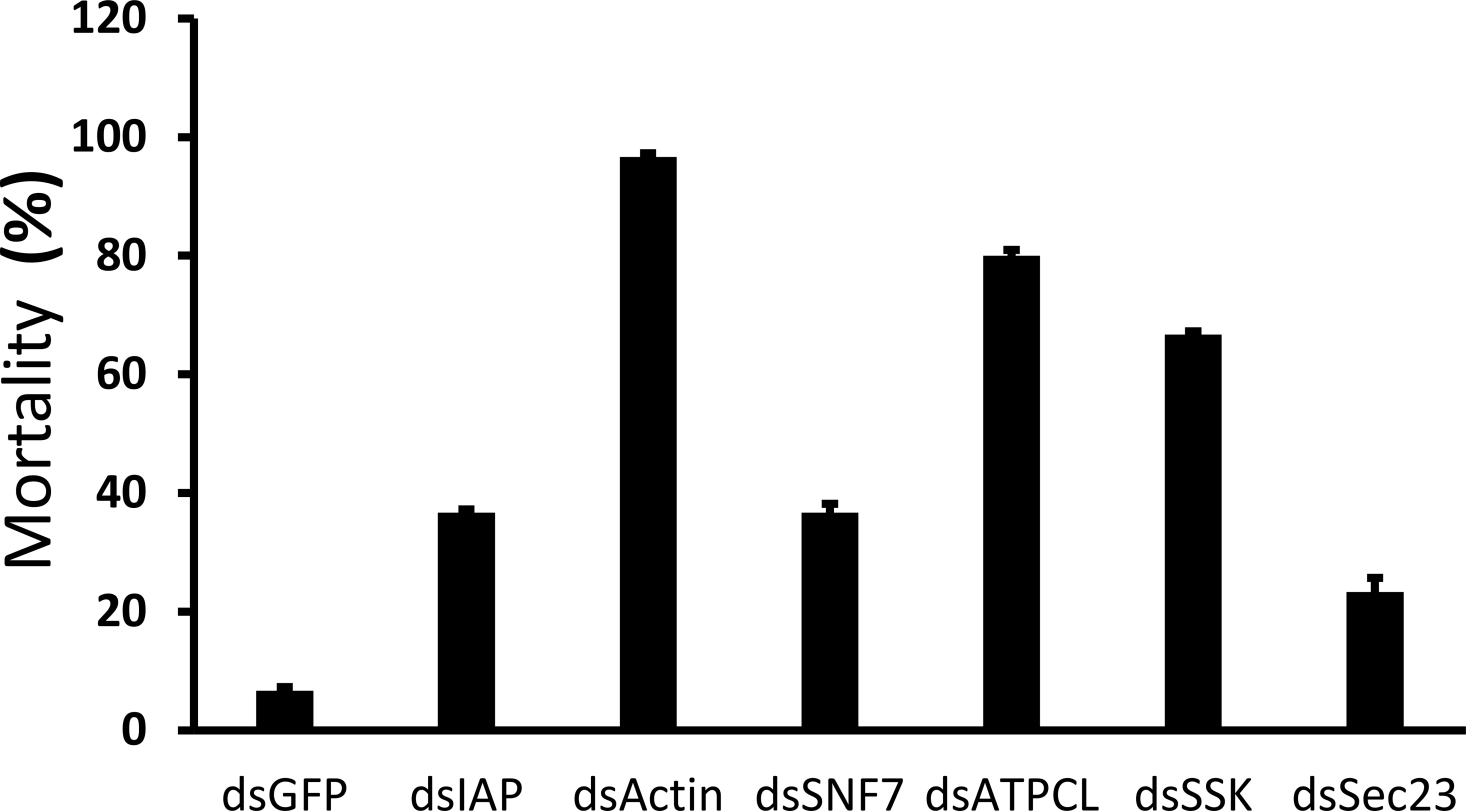
Identification of RNAi target genes in Japanese beetle adults. Ten micrograms of dsRNA targeting each gene were injected into the beetle. Mortality was assessed on the 10^th^ day after dsRNA injection. dsGFP was used as a control. The experiment was repeated 3 times. Mean ± S.D (N = 30) are shown.

**Figure 2:**
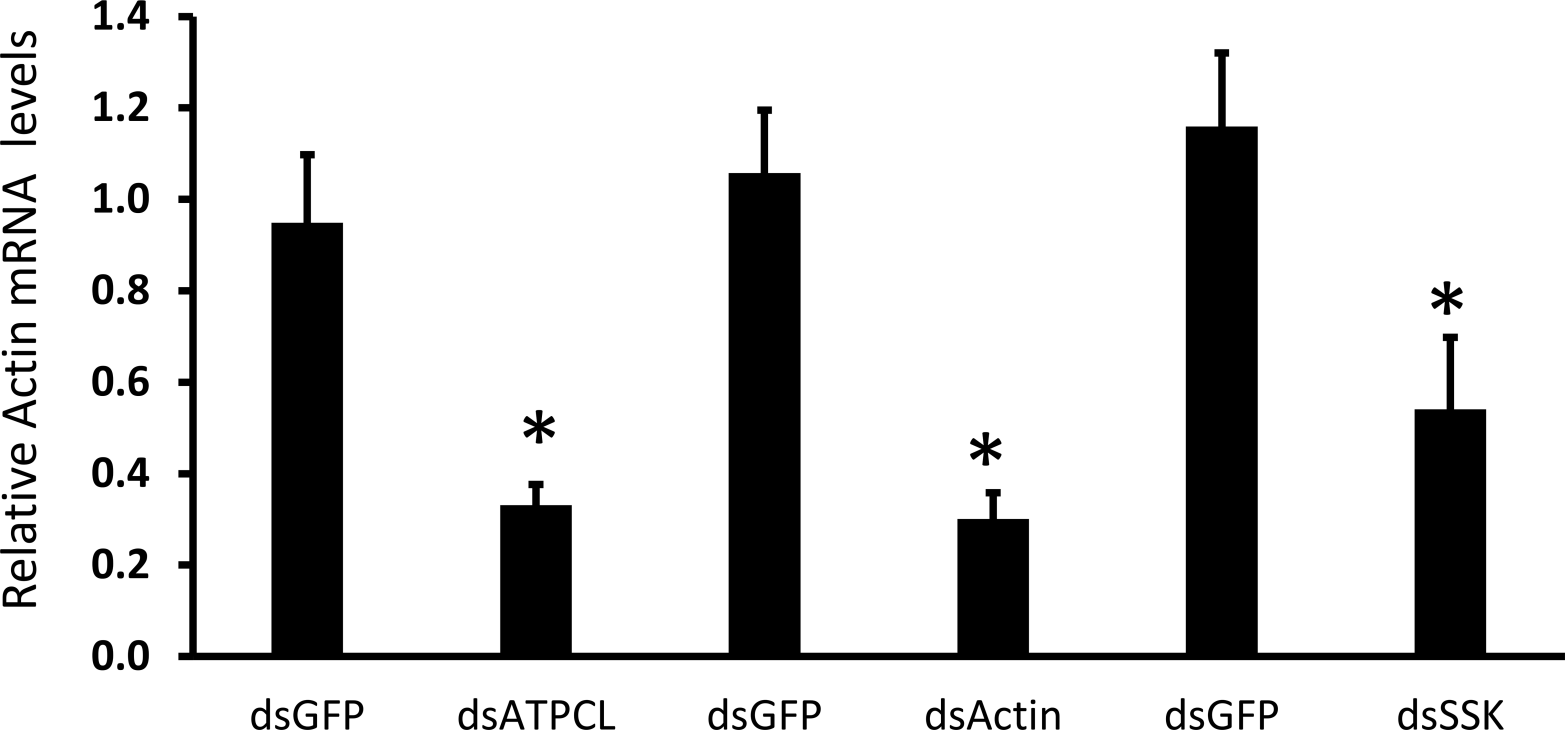
dsRNAs induce knockdown of target gene expression in JB adults. Adults were injected with 10 µg of dsATPCL, dsActin, dsSSK, or dsGFP (control). Total RNA was extracted from adults on the 5^th^ day after injection, and relative mRNA levels of target genes were determined using RT-qPCR. mRNA levels. The relative mRNA levels were normalized using RPL32 as a reference gene. Mean ± SE (N = 5) are shown. Statistical significance was assessed using one-way ANOVA with a Tukey *post hoc* test. An asterisk indicates a significant difference between the control and treatment groups (P < 0.05).

To identify target genes for RNAi-mediated control of JB, 10 µg of dsRNA targeting each of the six selected genes was injected into ten JB adults, and mortality was recorded on the 10^th^ day post-injection. Injection of dsRNA targeting three genes caused more than 70% mortality. The dsActin induced nearly 100% mortality within 7 days, whereas control insects injected with dsGFP showed less than 10% mortality. dsIAP and dsSNF7 caused 30% and 20% mortality, respectively (**Figure 1**). We selected three genes (ATPCL, Actin, and SSK) for assessing knockdown efficiency. Injection of dsATPCL, dsActin, and dsSSK into JB adults caused 63, 72, and 54 percent reduction in mRNA levels, respectively, when compared to their levels in control adults treated with dsGFP (**Figure 2**).

Finding suitable target genes is important for successful RNAi-mediated control in JB adults. In this study, out of the dsRNAs targeting six genes screened, three showed higher mortality in JB adults. In contrast, targeting three other genes (IAP, SNF7, and Sec23) did not cause significant mortality. dsRNAs targeting the IAP gene have been reported to cause lethality across different insect species (17–20), but did not perform well in JB. This may be attributed to differences in gene paralogs with overlapping functions, gene transcription patterns and dsRNA concentrations used, among other factors (8, 21–25). This suggests that targeting orthologous genes with dsRNA may not elicit the same phenotype across insects. Therefore, screening orthologous genes of known RNAi targets could be useful for identifying target genes in the pest species of interest. Silencing the Actin gene showed higher mortality than targeting the other genes. The Actin gene is involved in cellular processes such as cell motility, cell division, and muscle contraction. In addition, knockdown of the Actin gene showed feeding inhibition and mortality in multiple insect species (12, 26–29). Our results demonstrated that targeting the Actin gene caused 100% mortality within 7 days in JB adults. dsATPCL and dsSSK also induced 80 and 70% mortality by 10 days post-injection. The 10% mortality observed in dsGFP-treated JB adults may be attributable to the length of the bioassay, as previously reported (30). These data suggest that the Actin gene may be a promising target for JB adult control.

### Oral delivery of dsRNA

**Figure 3:**
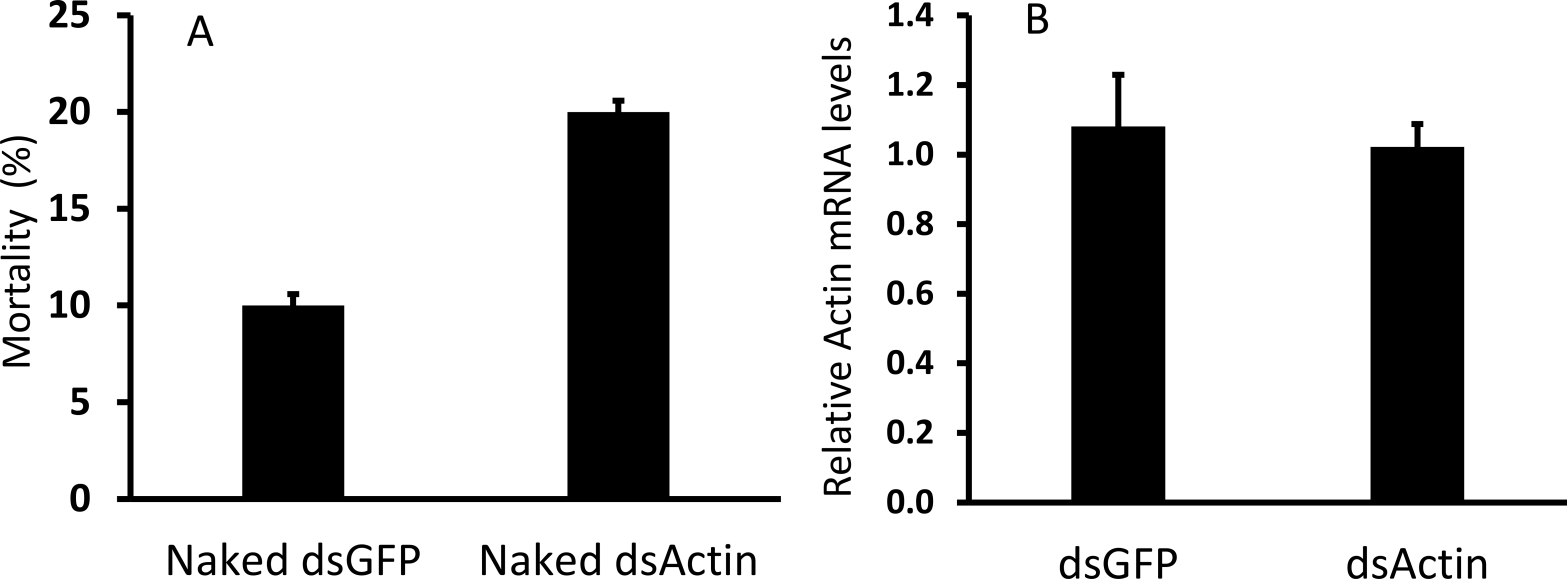
**(A)** JB adult mortality was assessed 15 days after feeding on naked dsRNA-coated linden leaves. 300 µg of dsRNA was dissolved in 2 mL of DI water and coated onto linden leaves. Mortality was recorded on the 15^th^ day post-feeding, and percent mortality was calculated. The experiment was repeated three times under the same conditions. (Mean ± S.D) (N = 30) are shown. **(B)** Naked dsRNA does not induce knockdown of the target gene in JB adults. Total RNA was isolated from JB adults fed on leaves coated with naked dsActin or dsGFP on the 5^th^ day post-feeding. Relative Actin mRNA levels were determined by RT-qPCR and normalized to RPL32. (Mean ± S.D) (N = 5) are shown.

**Figure 4:**
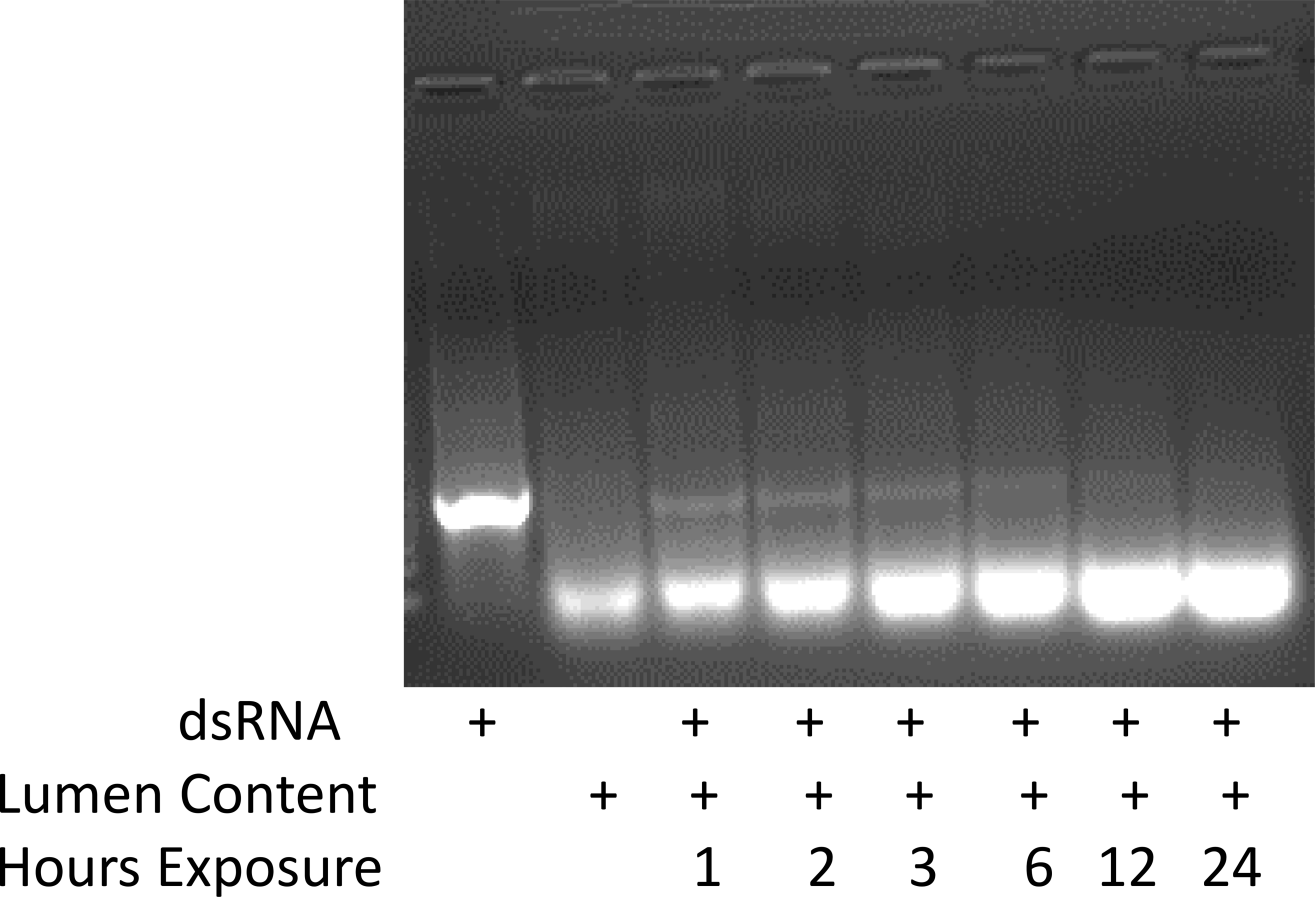
Assessing the stability of dsRNA in the midgut lumen contents of JB adults. One microgram of dsRNA was incubated with one microgram of lumen content protein at different points (1, 2, 3, 6, 12, and 24 hr), the samples were run on a 1% agarose gel, stained with GelRed, and photographed using an iBright™ CL1500 Imaging System (ThermoFisher Scientific, USA).

Oral delivery of dsRNA could be a useful method for controlling JB adults. If this approach is effective, it will open a new avenue for insect pest control. Therefore, we sought to develop an oral delivery system for dsRNA by feeding dsActin to adult JB. Feeding naked dsActin did not cause significant mortality in JB adults (**Figure 3A**). We also assessed knockdown efficiency on 5th day after feeding. The results showed that feeding naked dsActin did not reduce expression of Actin gene in JB adults (**Figure 3B**). We hypothesized that this might be due to degradation of dsRNA in the midgut lumen JB. To determine whether the dsRNA degradation was due to nucleases present in the midgut lumen contents, one microgram of dsRNA was incubated with 1 µg of lumen contents for different lengths of time (1 to 24 hr). The results showed that midgut lumen contents started degrading dsRNA within 12 hr of exposure, and most of the dsRNA was degraded by 24 hr after exposure (**Figure 4**). These results suggest that the nucleases present in the adult JB midgut lumen could degrade ingested dsRNA. This may be one reason why feeding naked dsRNA does not induce RNAi in JB adults.

### dsActin expressed in *E. coli* kills JB adults

**Figure 5:**
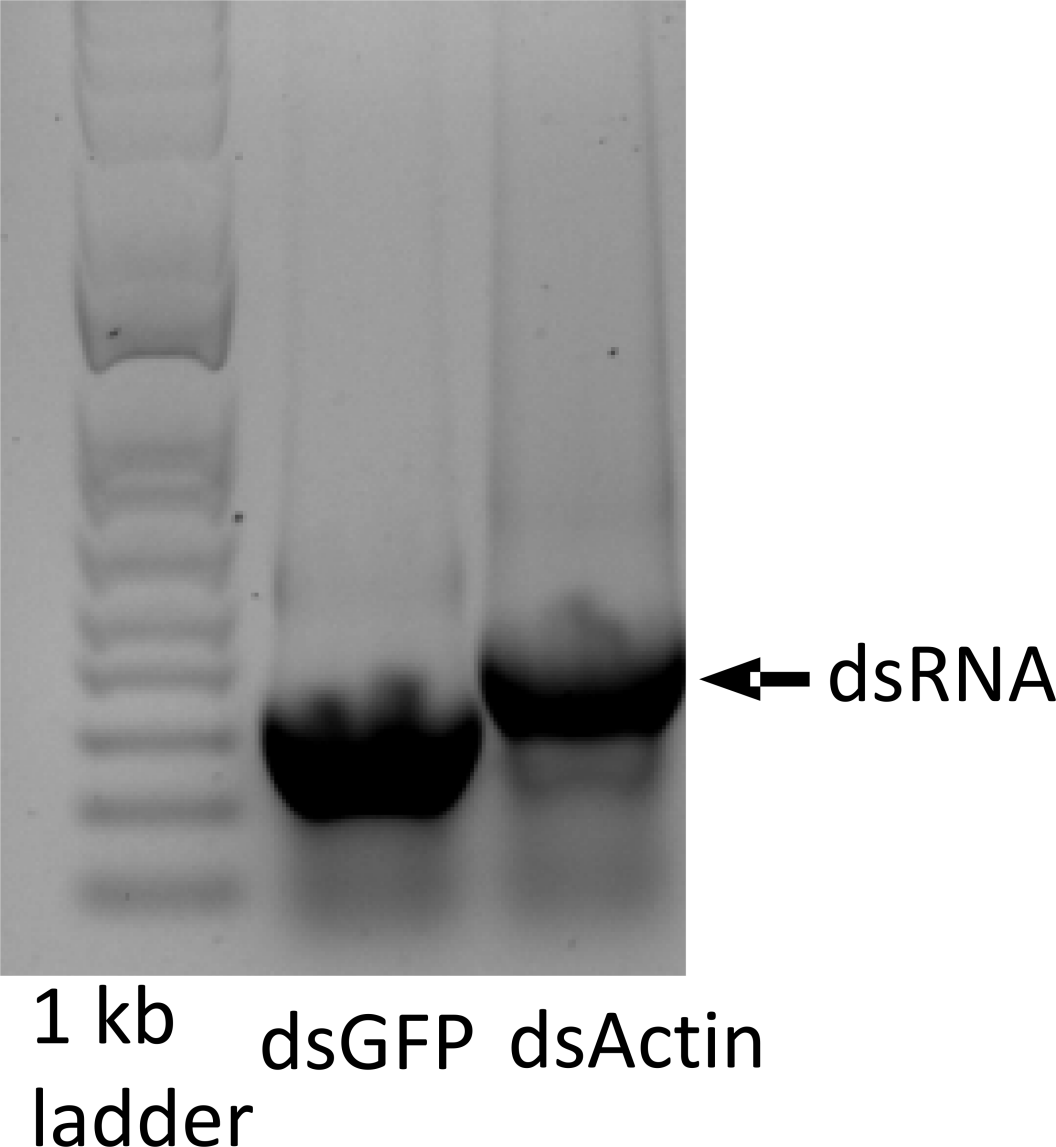
*E. coli* cells with L4441 plasmid containing Actin and GFP target sequences produce dsActin and dsGFP. The bacteria (HT115 DE3) were cultured in the medium containing IPTG, RNA was extracted from 1 ml of culture of bacteria and run on a 1% agarose gel. The photograph of the stained gel is shown.

**Figure 6:**
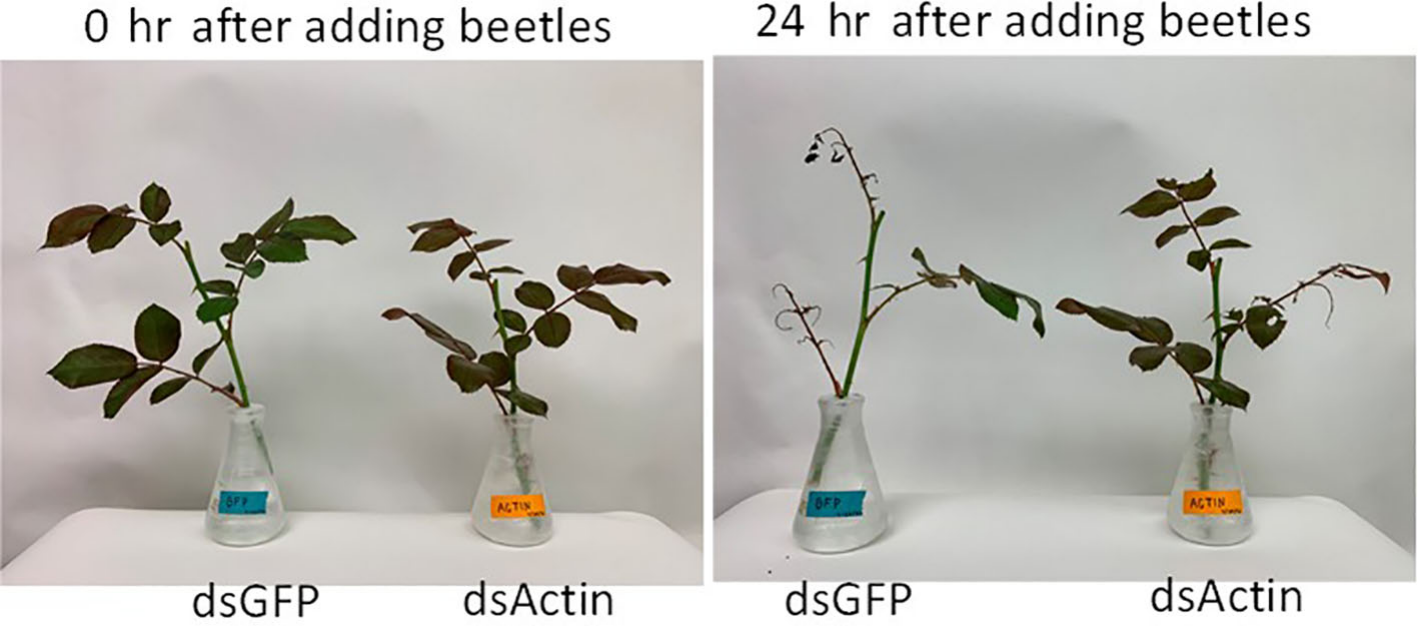
Bacteria produced orally delivered dsRNA induces feeding inhibition in JB adults. P. Japonica dsActin and dsGFP (control) produced in bacteria were sprayed on rose leaves and fed to P. Japonica. Six days after dsRNA treatment, ten P. Japonica beetle adult females were released into each cage, where a fresh branch of rose shrub was placed. Photographs were taken at 0 and 24 hr after the beetles were added. The dsGFP-treated beetles consumed most of the leaves, while the dsActin-treated beetles consumed only a couple of leaves, suggesting that treatment with dsActin induces feeding inhibition.

**Figure 7:**
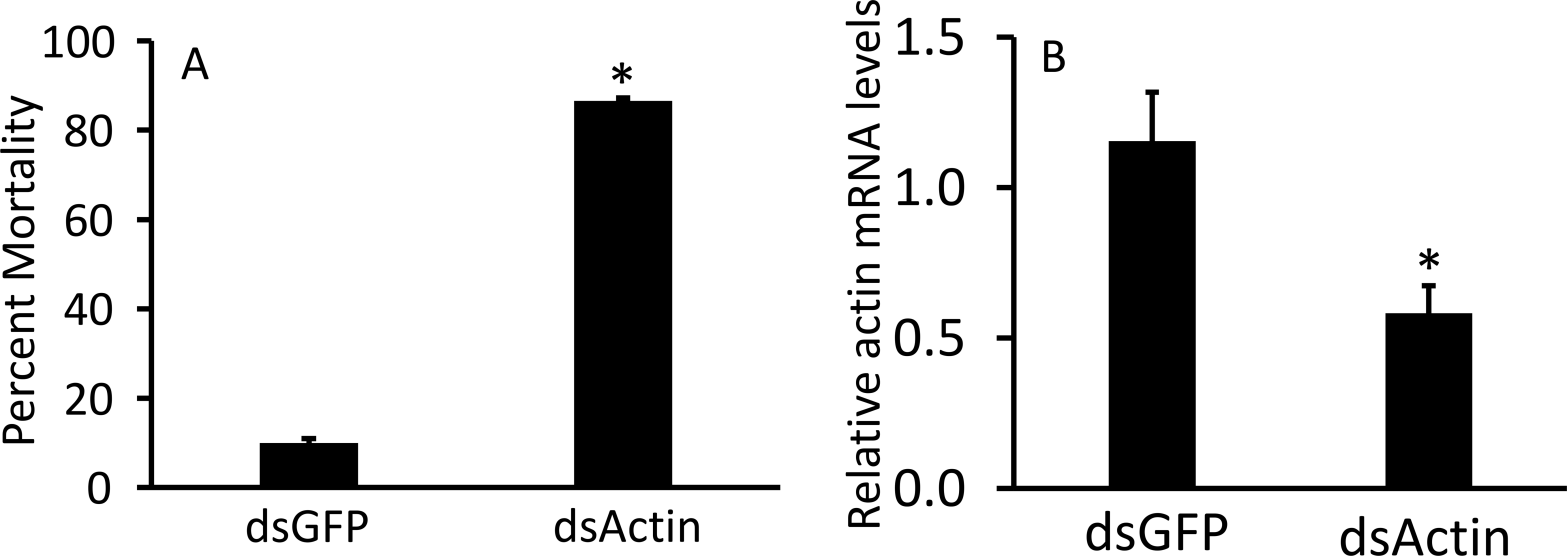
RNAi induced by heat-killed bacteria expressing dsActin. **(A)** bacterial culture (100 mL) was centrifuged at 2500 rpm for 10 minutes at 4 °C. The supernatant was discarded, and the pellet was washed with 1× PBS. The pellet was resuspended in 2 mL of double-distilled water and incubated at 80 °C for 30 minutes. 2 mL of inactivated bacteria was sprayed on Rose leaves; mortality was recorded on the 6^th^ day post-feeding, and percent mortality was calculated. The experiment was repeated three times under the same conditions. (Mean ± S.D) (N = 30). **(B)** RNAi induced by heat-killed bacteria in Japanese beetles, as measured by a feeding bioassay. The mRNA levels were determined on the 3rd day after feeding using RT-qPCR. Relative mRNA levels were normalized using RPL32 as a reference gene. Mean ± S.E. (n = 5) are shown. Statistical significance was assessed using one-way ANOVA with a Tukey *post hoc* test. An asterisk indicates a significant difference between the control and treatment groups (P < 0.05).

To protect dsRNA from nuclease degradation, different approaches, including nanoformulations (31–33), expression in chloroplasts (29, 34), yeast (35) and bacteria (17, 36, 37) have been reported. Among them, the bacterial expression system can be readily scaled up and is cost-effective. We selected the *Actin* gene because of the higher knockdown efficiency and mortality observed after treating adult JB with dsActin. The dsActin was produced using RNase III-deficient *E. coli* cells. The transformed bacteria were induced with IPTG to overexpress dsRNA, which was confirmed by agarose gel electrophoresis (**Figure 5**). To test the insecticidal activity of inactivated bacteria against JB adults, rose (*Rosa* sp.) leaves were sprayed with inactivated bacteria expressing dsActin or dsGFP (control). Then the leaves were fed to the adult beetles. We observed reduced feeding activity of JB on plants treated with dsActin compared to those treated with dsGFP as a control. (**Figure 6**). More than 90% mortality was detected in beetles feeding on rose leaves treated with dsActin compared to about 10% mortality observed in beetles feeding on dsGFP-treated leaves (**Figure 7A**). These results indicated that applying inactivated bacteria expressing dsActin could protect rose plants from JB. To confirm that bacterially expressed dsRNA was delivered to beetles via feeding and to verify knockdown of the target gene, we measured Actin mRNA levels in beetles fed on dsActin- or dsGFP-treated leaves. After three days of feeding, the beetles were collected, and Actin mRNA levels were measured using RT-qPCR. The results showed that the Actin mRNA levels are significantly decreased in JB adults fed on dsActin compared to the levels in control dsGFP-fed JB (**Figure 7B**). These results demonstrate that the dsActin produced in bacteria was indeed ingested by beetles, resulting in knockdown of target gene expression. These results also demonstrate that bacterial proteins and other macromolecules protect dsRNA from nuclease degradation in the gut lumen and facilitate its uptake and transport into cells.

### Field testing of dsRNA

**Figure 8:**
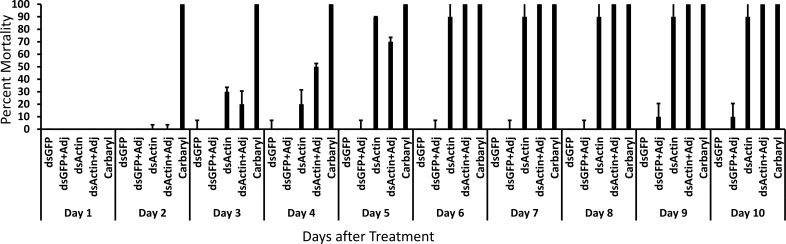
dsGFP, dsActin, dsGFP plus adjuvants, dsActin plus adjuvants, and carbaryl as a control, prepared for the field trials, were evaluated in the greenhouse by spraying rose plants and assessing their effect on JB released onto these plants. Mortality was recorded over a 10-day period. Ten beetles were used in each replicate, and each treatment had three replicates. Mean + S.E (N = 10) are shown.

We tested five treatments: 1. heat-killed bacterial extract containing dsGFP, 2. heat-killed bacterial extract containing dsActin, 3. heat-killed bacterial extract containing dsActin, each mixed with 1% adjuvants, NuFilm P (spreader-sticker) and 1% Monterrey Horticultural Oil (penetrator), 4. Carbaryl insecticide as a positive control and 5. untreated plants as a negative control. The materials prepared for field experiments were tested in JB adult bioassays. The positive control, Carbaryl, caused 100% mortality by Day 2 after application (**Figure 8**). The dsGFP-expressing bacterial extract alone or mixed with adjuvants caused less than 10% mortality by Day 10 after treatment. The bacterial extract containing dsActin alone or when mixed with adjuvants, induced approximately 30% mortality in beetles by Day 3, and both treatments reached 100% mortality by Day 6. These results showed that the materials prepared function as expected and therefore could be used in field experiments. As expected, dsActin acted more slowly than the insecticide, carbaryl.

### dsActin treatment reduced JB population, protected okra plants from defoliation, and increased fruit yield in netted plants, but not in the open field

**Figure 9:**
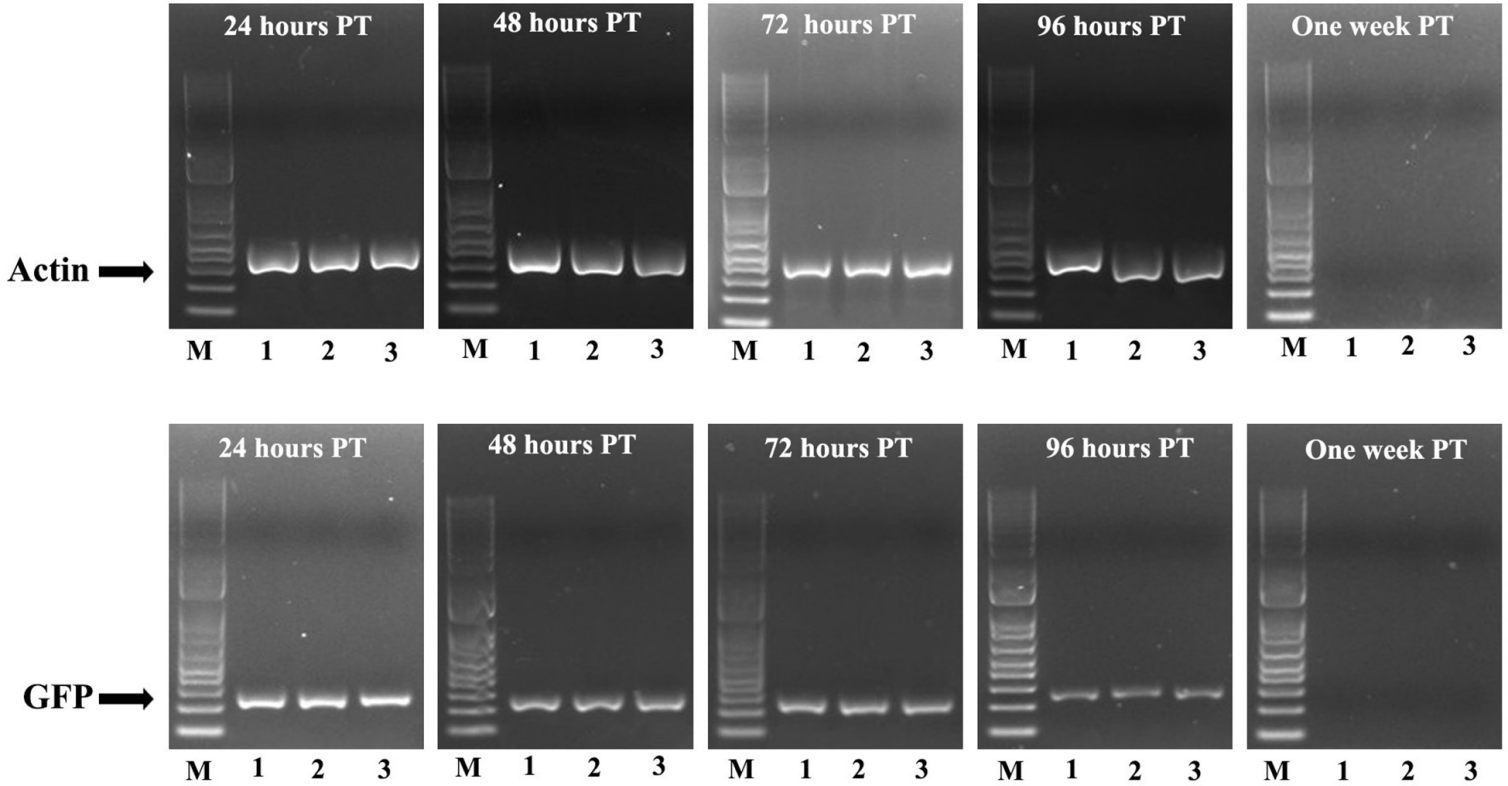
Leaves collected from treated plots at 24, 48, 72, 96 hours, and one week after treatment were used to isolate RNA. The RNA was converted to cDNA and used in RT-PCR to detect dsActin and dsGFP. The PCR products, along with a 1 kb ladder, were run on agarose gels, stained with GelRed, and photographed using an iBright™ CL1500 Imaging System (ThermoFisher Scientific, USA).

**Figure 10:**
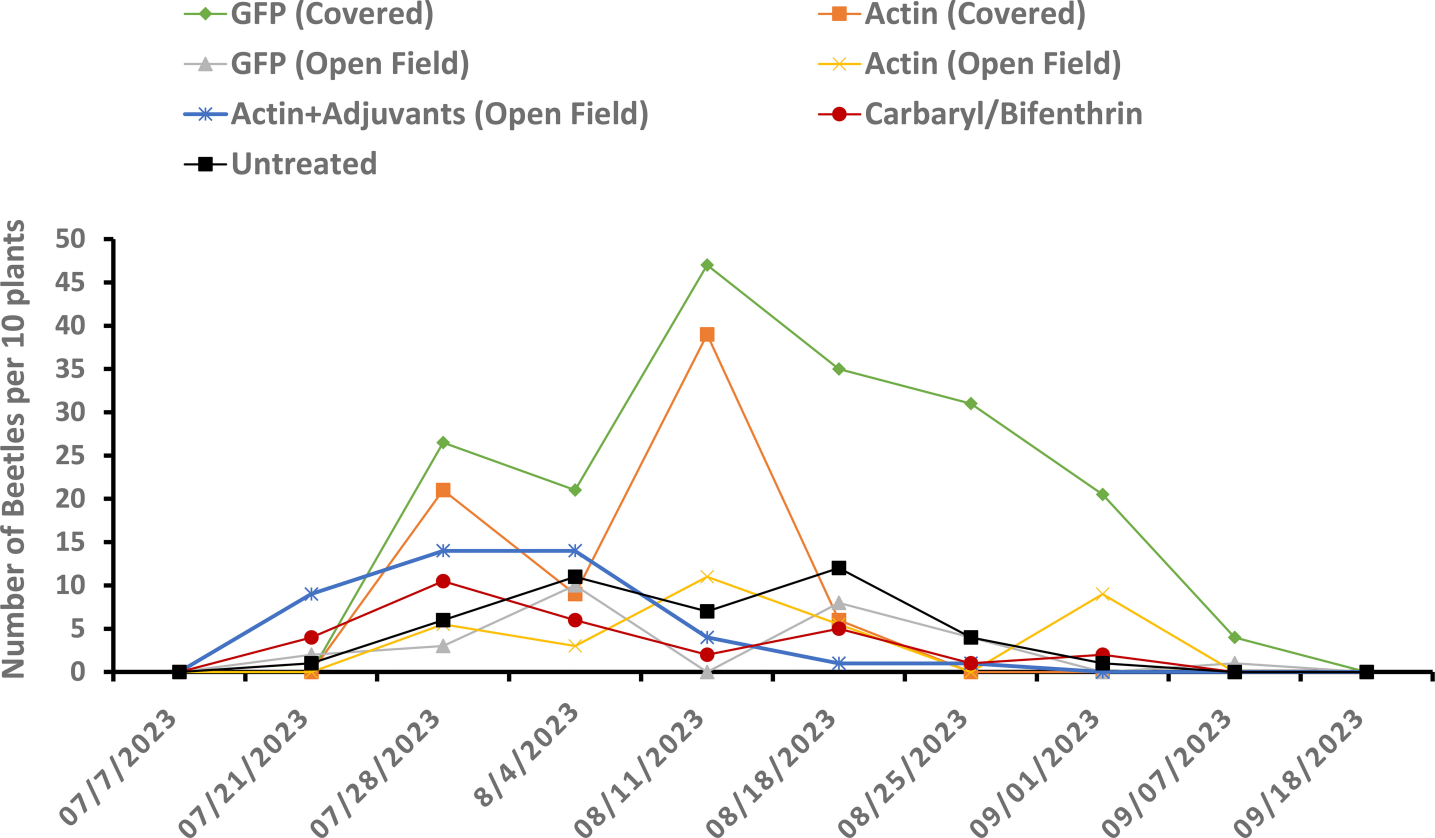
Number of beetles found in plots. Each plant was visually assessed by two observers weekly at the same time of day to confirm accurate counts of all adult JB in each plot. The total number of JB adults found on 10 plants in each treatment are shown.

**Figure 11:**
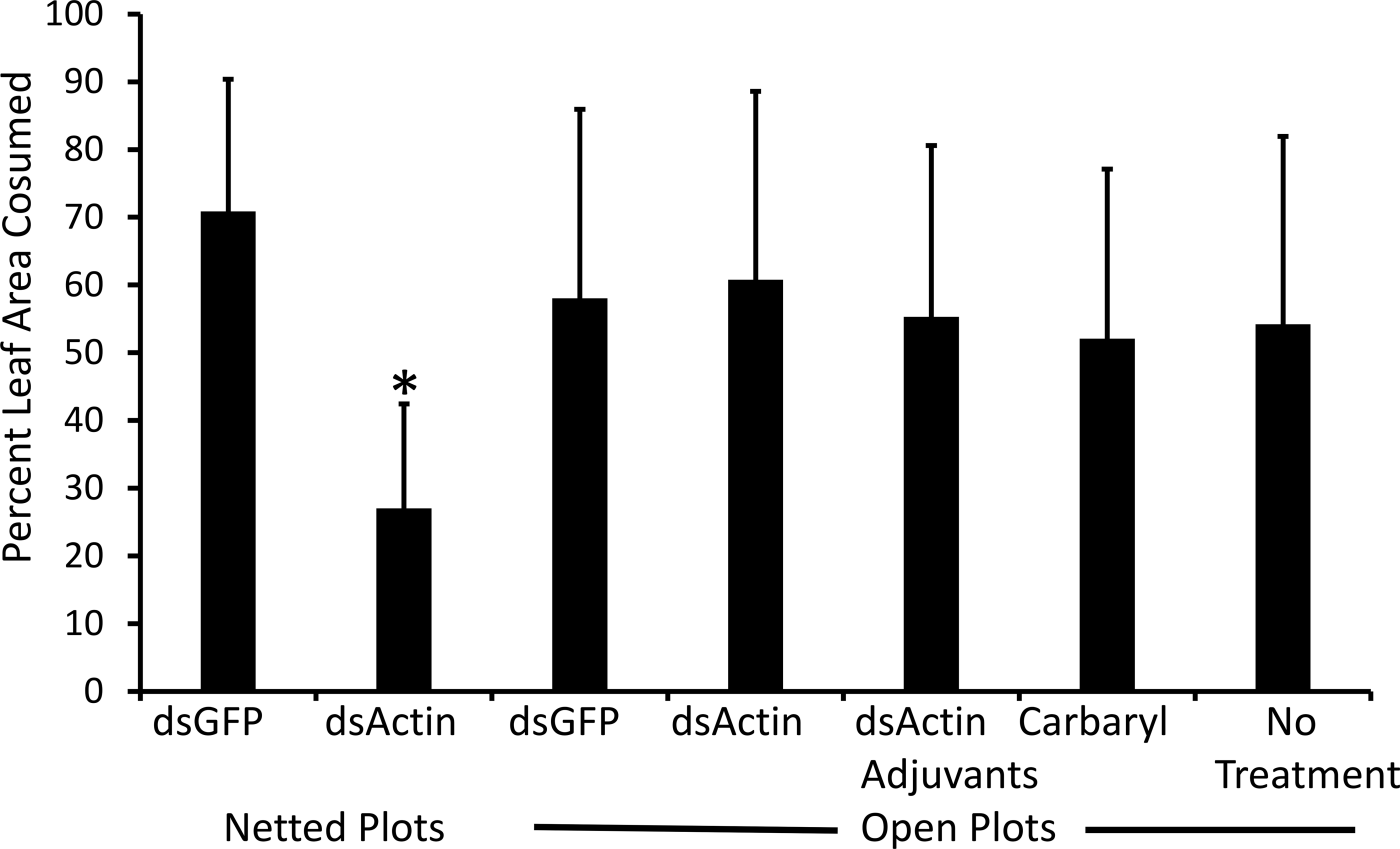
dsActin produced in bacteria and sprayed on okra plants protects them from damage. The five uppermost leaves from each plant within a given plot (50 leaves per plot) were removed and scanned to determine the average damage by JB adults using computer software (ImageJ and LeafByte. Statistical significance was assessed using one-way ANOVA with a Tukey *post hoc* test. An asterisk indicates a significant difference between the control and treatment groups (P < 0.05).

**Figure 12:**
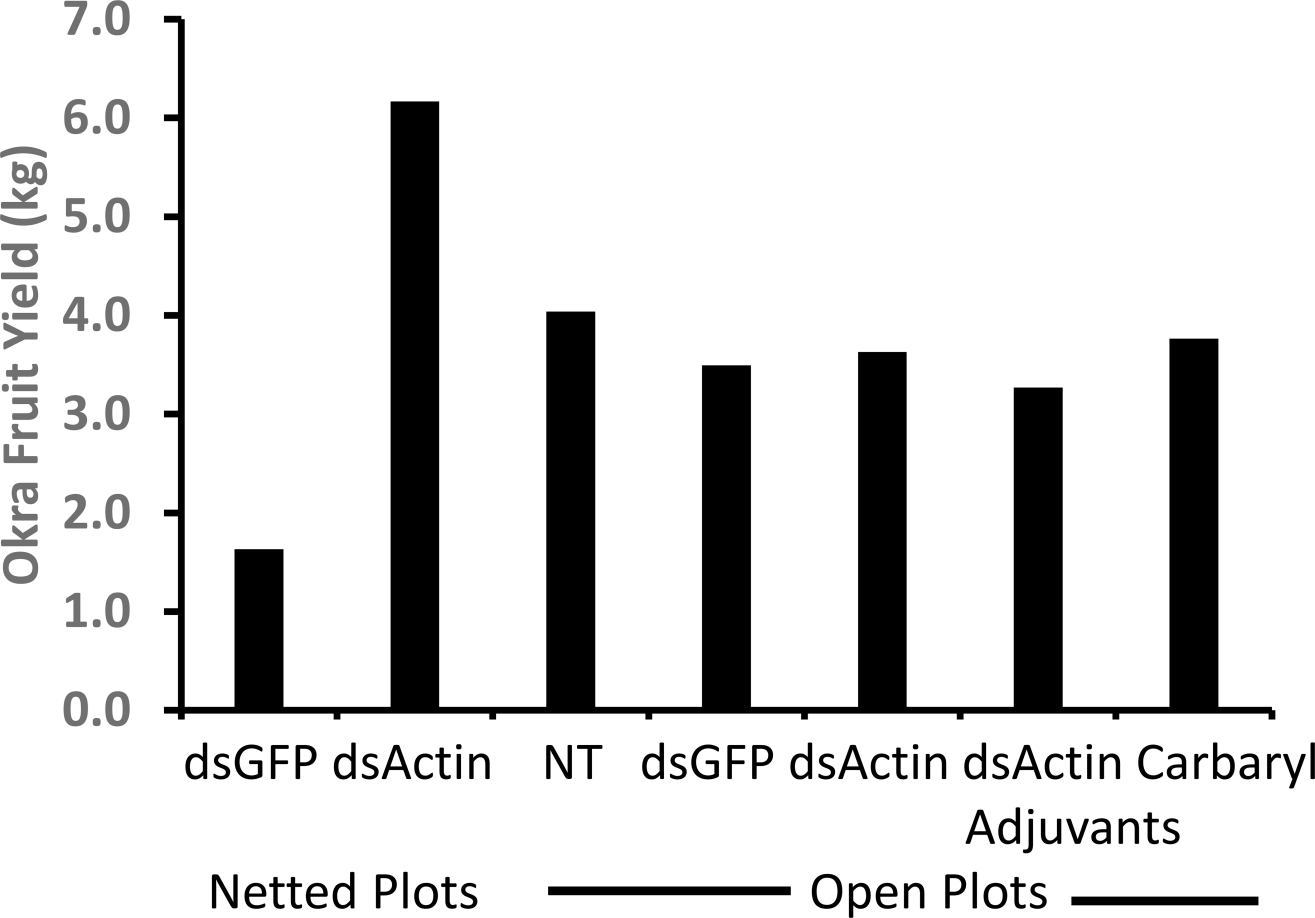
Increase in okra fruit yield in dsActin-treated covered plants compared to that in dsGFP-treated covered plants and all other treatments in the open field. Okra fruit was harvested from the ten plants in each treatment, and the total weights of okra fruit from 10 plants from each treatment are shown.). NT, no treatment.

The dsRNAs were sprayed onto okra plants. Five treatments were applied to plants in an open field, and an additional two treatments (dsGFP and dsActin bacterial extracts) were applied to plants enclosed in nets. Stability of dsRNA on leaves, JB numbers in each plot, leaf damage, and okra fruit yield were recorded. Total RNA isolated from okra leaves collected from plants sprayed with dsGFP and dsActin was converted to cDNA, and primers designed based on GFP and Actin gene fragments targeted by their respective dsRNAs were used in PCR. PCR products run on agarose gels showed the presence of both dsGFP and dsActin until 96 hr after treatment, but not at one week after treatment (**Figure 9**). JB numbers were similar across all treatments, including the no-treatment control, in plots not covered with nets (**Figure 10**). However, the dsActin bacterial extract showed a lower number of beetles compared to dsGFP-treated plants. The initial increase in beetle numbers in covered plots is associated with the release of 100 beetles per plot. The average leaf damage was similar in all treatments in open field plots was not significantly different. In the covered plots, leaf damage was lower in dsActin-treated plots than in dsGFP-treated plots (**Figure 11**). Also, the yield of okra fruits was similar in all treatments in open field plots, but in the covered plots, fruit yield was higher in dsActin-treated plots than in dsGFP-treated plots (**Figure 12**). These results suggest that dsActin can protect okra plants from defoliation and increase yield. The movement of JB among plants sprayed with different treatments may have contributed to the lack of differences in defoliation and fruit yield observed among these treatments.

The JB is a major invasive pest that damages both turf landscapes and crops. Traditional approaches to controlling this insect can harm the environment and beneficial species; therefore, there is an urgent need to develop highly targeted, environmentally safe methods, such as RNAi, to control this pest. The results of our studies demonstrate that applying heat-inactivated bacteria expressing JB dsActin to the surface of host plant leaves results in significant feeding inhibition and JB adult mortality. These results inform product development aimed at managing this serious pest. The field experiments showed that dsActin produced in E. coli could be used to control JB; however, further formulation improvements are needed to increase dsRNA stability under field conditions.

Improving the dsRNA product for JB control also requires further research. Because of the active movement of JB adults between plots, we did not detect differences in treatment effects in open plots, including the dsActin treatment. In contrast, dsActin performed better than dsGFP in killing JB, protecting okra plants, and increasing fruit yield. Future studies will incorporate information on JB movement and space treatments to determine the minimum distance between farms required for effective use of dsActin as an insecticide to manage JB populations. Nevertheless, our results suggest that dsActin could be a suitable choice for area-wide control of JB on large farms. This could also be used to control JB near airports, thereby preventing the spread to regions free of JB.

## Data Availability

The original contributions presented in the study are included in the article/**Supplementary Material**. Further inquiries can be directed to the corresponding author.
