# Supplementary material for: Evaluation of dsRNA produced in E. coli for controlling Japanese beetle
Source: Front Insect Sci. 2026 Apr 29;6:1811042. doi: 10.3389/finsc.2026.1811042 (PMC13167589; doi:10.3389/finsc.2026.1811042)
Supplement: Supplementary file 1 [file SupplementaryFile1.docx]

**Supplementary Information**

Table S1. Primers used in the Studies

| Primer name | Primer sequence (5’ to 3’)* |
| --- | --- |
| F-JBdsActin  R-JBdsActin | CATGGTCGGTATGGGTCAAA  TCAGCCGTAGTTGTGAATGAG |
| F-JBdsSec23  R-JBdsSec23 | TTCCGTTAGGGTGCCTATATC  ACCTAATTCTTCGTCGTCCATACˈ |
| F-JBdsSnf7  R-JBdsSnf7 | AATCCGATAAGGGCCCAACˈ  CCTCTCGTTGCATTTCAATAGˈ |
| F-JBdsSSK  R-JBdsSSK | TACACTATTGGCACGGGTATTT  AATCTATTCCTCTTCCGTCAGˈ |
| F-JBdsIAP  R-JBdsIAP | GCCAATTAGAGGTCGGCTATTˈ  TTCTTCCCACGGTTCATCAC |
| F-JBdsATPCL  R-JBdsATPCL | CCACCGGCAAAGAACTAATAAAC  ATGAACGGCTCTATGATGAAˈ |
| F-JBActinqPCR  R-JBActinqPCR | GACCAACAATGGATGGGAAGAˈ  CGTAGACAATGGATCCGGTATG |
| F-JBATPCLqPCR  R-JBATPCLqPCR | CGATCAGCGAGTGAAGATTGT  GGCTTCTTGCTCGTGGTAAT |
| F-JBSSKqPCR  R-JBSSKqPCR | AGAGAGCTCGAGCAGTTATAGA  AGGGTTTGAAGCCACCATATAA |
| F-JBRPL32qPCR  R-JBRPL32qPCR | CCATATGCTCCCTACTGGTTTC  CGCAATTTCACCGCAGTATTT |

*T7 promoter sequence, TAATACGACTCACTATAGGG, was added at the 5’ end of each primer used in amplification of dsRNA template.


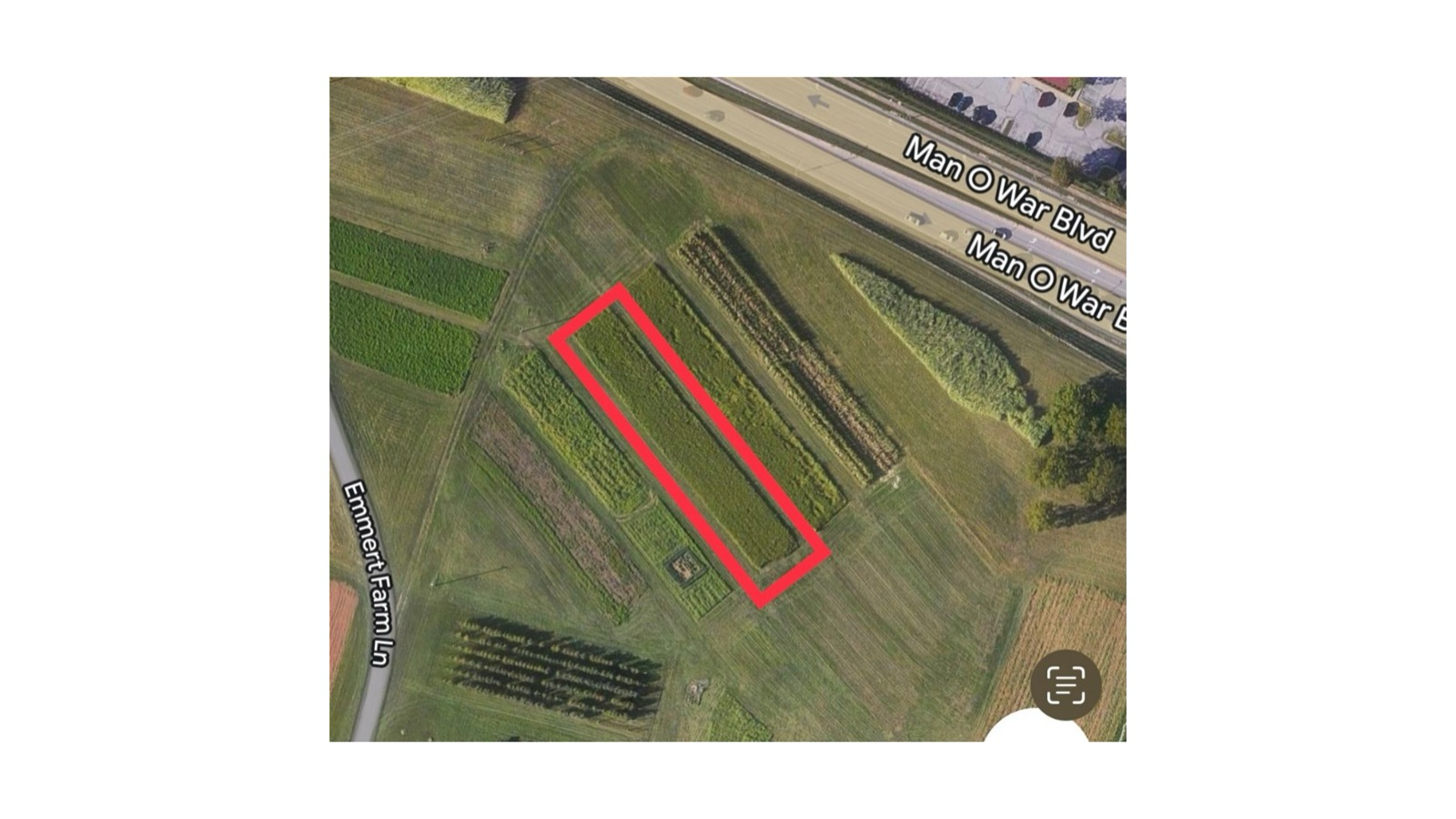


Figure S1 The area used for field training in the University of Kentucky South Farm is marked with a red rectangle.


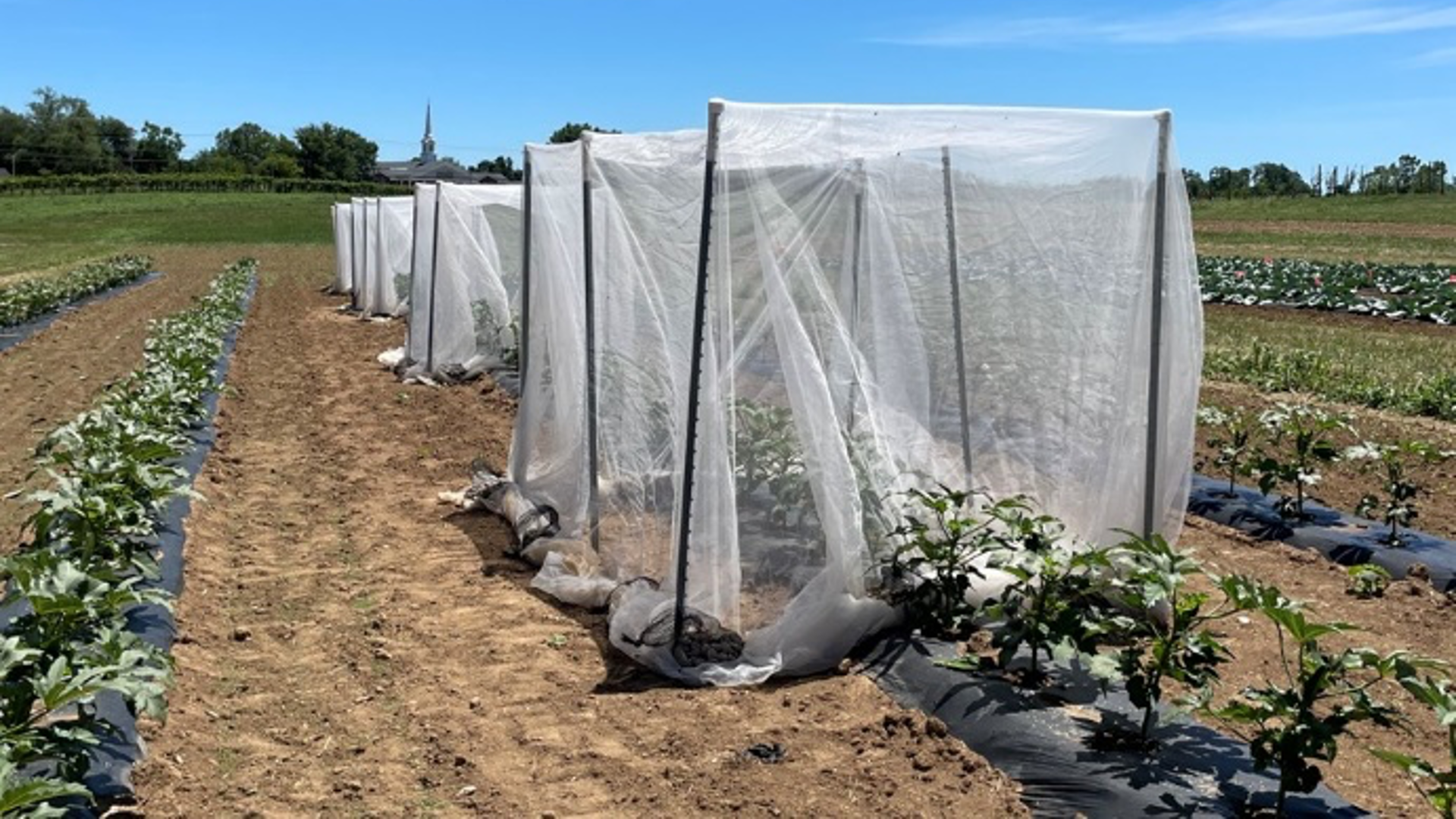


**Figure S2**. Pictures of both covered and open okra plants used in the studies.
